# Development of a novel gout treatment patient decision aid by patient and physician: A qualitative research study

**DOI:** 10.1111/hex.13184

**Published:** 2021-01-12

**Authors:** Meykkumar Meyappan, Wei Siong Aaron Loh, Li Yen Tan, Sheng Feng Ian Tan, Pey Ying Ho, Yih Jia Poh, Ngiap Chuan Tan

**Affiliations:** ^1^ SingHealth Polyclinics Singapore City Singapore; ^2^ Yong Loo Lin School of Medicine National University of Singapore Singapore City Singapore; ^3^ Rheumatology Department Singapore General Hospital Singapore City Singapore; ^4^ SingHealth‐Duke NUS Family Medicine Academic Clinical Programme Singapore City Singapore

**Keywords:** gout, patient, patient decision aid, physician, shared decision making, treatment

## Abstract

**Background:**

Gout treatment is not optimized globally, often due to therapeutic inertia by physicians or poor adherence to urate‐lowering medications by patients. A patient decision aid (PDA) to facilitate shared decision making (SDM) in gout treatment may overcome these physician‐patient barriers.

**Objective:**

The study explored the views of physicians and patients on a novel locally designed gout treatment PDA prototype.

**Design:**

Qualitative descriptive design was used to gather data from in‐depth‐interviews (IDI) and focus group discussions (FGD). Data analysis was via thematic analysis. Emergent themes shaped a revised version of the PDA.

**Setting and participants:**

Adult Asian patients with recent acute gout exacerbations and local Primary Care Physicians (PCP) in Singapore were purposefully chosen. 15 patients with gout and 11 PCPs participated across three IDIs and six FGDs, with the investigators exploring their views of a prototype gout treatment PDA.

**Results:**

Patients and physicians generally concurred with the content and design of the PDA prototype. However, while patients preferred fewer treatment details, the PCPs desired more information. Patients preferred the display of statistics, while PCPs felt that numbers were not relevant to patients. The latter were hesitant to include treatment options that were unavailable in primary care. Both stakeholders indicated that they would use the PDA during a consultation. PCPs would need further training in SDM, given a lack of understanding of it.

**Conclusion and patient contribution:**

Patients will be the prime users of the PDA. While their views differed partially from the physicians, both have jointly developed the novel gout treatment PDA.

## 1 INTRODUCTION

The global disease burden of gout has been rising.^1^ Poorly controlled gout can lead to gouty tophi, joint deformities with functional loss, poor physical,^2^, ^3^, ^4^, ^5^ psychological,^2^, ^5^, ^6^, ^7^ social^2^, ^6^ and financial outcomes,^3^, ^4^ that adversely impact the quality of life.^3^, ^4^

Despite such significant morbidity, gout is not optimally managed globally. Worldwide studies have shown that failure to reach target serum uric acid levels occurs in large proportions of patients with gout.^8^, ^9^, ^10^, ^11^, ^12^, ^13^ Unpublished local data showed that only half of patients with frequent recurrent acute gout exacerbations were treated with Allopurinol. Amongst those treated, only half were consistently taking the Allopurinol. Similar results were reported in a tertiary rheumatology clinic in Singapore.^14^

Suboptimal management of gout is multifactorial, involving both patient and physician.^15^, ^16^, ^17^, ^18^ Poor patient adherence to urate‐lowering therapy (ULT) ranged from 10% to 46%.^19^ This is below the World Health Organization's estimate that 50% of adults adhere to long‐term therapy in other chronic diseases.^20^ Reasons for poor medication adherence included a lack of understanding of the need for ULT, belief that the treatment is ineffective, perceived prevalent adverse events with medication use and dissatisfaction with care from health‐care professionals.^16^, ^21^, ^22^ Physician factors included limited knowledge of gout and available gout treatment options, frustration with patients' poor medication adherence and time constraint during consultations.^15^, ^16^, ^18^

These barriers can potentially be addressed by the use of a patient decision aid (PDA) on gout and its treatment. A PDA is a tool to support decision making in selecting treatment options with due consideration to the user's values and preferences. It provides updated, evidence‐based and balanced information on the merits and risks of the treatment options and facilitates shared decision making (SDM) between patients and their physicians. By enhancing the understanding of the medical condition and therapy, PDAs have been shown to increase patient medication adherence, communication with their physician and trust towards the physician.^23^, ^24^, ^25^

A PDA on gout and treatment is lacking, which will cater to the multiethnic Asian population in an urban community such as Singapore, where gout is prevalent. At least 4.1% of adults in Singapore suffer from gout.^26^ This necessitates the development of a de novo PDA on gout and its treatment. Refinement with input from both patients and primary care physicians (PCPs) is an essential step to optimize its utility by these key users. A local gout treatment PDA prototype was thus created by a team of multidisciplinary health‐care professionals.

The study, therefore, aimed to gather feedback from patients and PCPs on the content, design, and perceived utility of a novel PDA prototype on gout and its treatment. It is a crucial step in the cultural optimization^27^ of the PDA, underpinned by conjoint efforts and contributions from both patients and health‐care professionals.

## 2 METHODS

### 2.1 Creation of a PDA prototype on gout and its treatment

**FIGURE 1:**
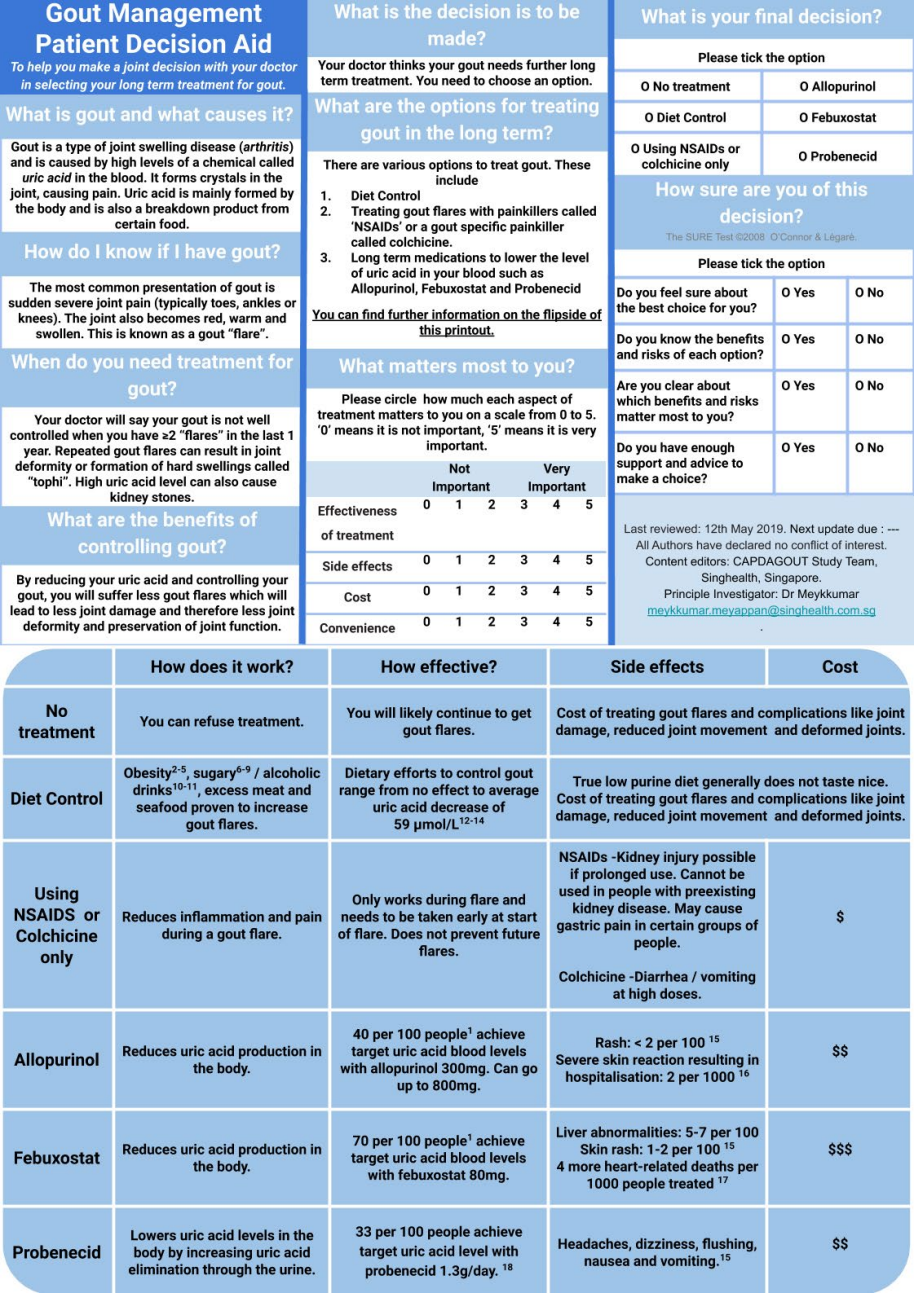
Front page and back page of prototype

**FIGURE 2:**
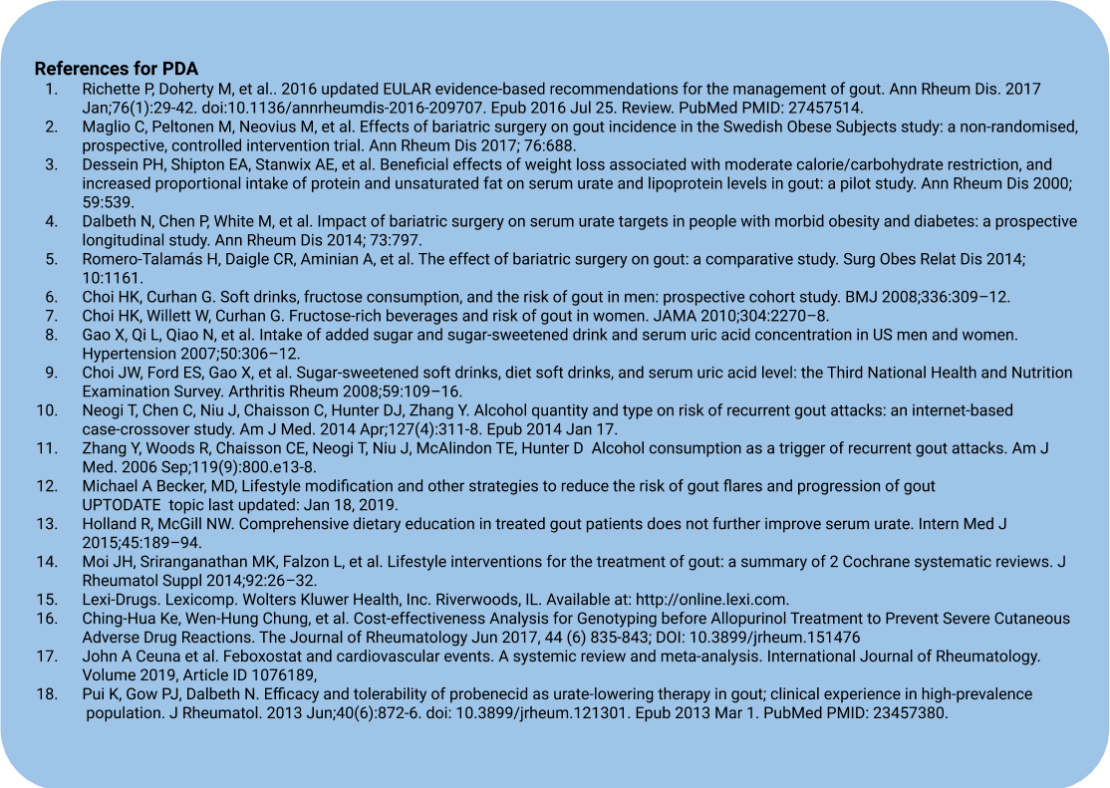
Optional Supplementary Page of DA references

Using the Ottawa decision support framework (ODSF) as a guide, a prototype gout treatment PDA (Figure 1 and Figure 2) was designed based on the latest evidence by a multidisciplinary team. This team comprised PCPs (MM, NCT), a rheumatologist (PYJ), a nurse (LYT), a pharmacist (IT), a dietician (PYH) and a medical student (AL).

Page 1 of the PDA covers basic knowledge of gout, indication for treatment, the decision to be made, options available, patient values, final decision to be made and the certainty of decision. Page 2 shows a table that compares the effectiveness, side‐effects and cost of the treatment options (Figure 1).

### 2.2 Conceptual framework

The Ottawa decision support framework (ODSF) is a conceptual framework based on concepts and theories from general psychology, social psychology, decision analysis, decisional conflict, social support and self‐efficacy.^28^ It is widely used in the development of PDAs. The Ottawa developmental process guided this PDA's development.^29^ The ODSF helped guide the ‘decisional needs’ of the user population to be elicited through the interviews and discussions. The ‘decisional needs’ of the patient include baseline knowledge of gout and treatment options, the prioritization of values in the decision to be made and the support and resources they tap on. Physicians must support patients through such decisions, and thus, their involvement under ‘decision support’ is critical.

### 2.3 Study design

A qualitative descriptive research approach was used.^30^ The in‐depth perspectives of the PDA from the patients and PCPs were expected to be subjective, perceptional, experiential and varied due to various contexts. One‐to‐one in‐depth interviews (IDI) and focus group discussions (FGD) were conducted to gather qualitative data.

### 2.4 Study site

This study was conducted in a typical public primary care clinic (polyclinic)^31^ in Pasir Ris estate, which serves a population of about 150 000 in northeast Singapore. Its 15 physicians and 28 nurses manage approximately 600 patients daily during office hours.

### 2.5 Participants

#### 2.5.1 Inclusion and exclusion criteria for patients

Adult multiethnic Asian adults, aged 21 years and above of both genders, with a clinical diagnosis of gout documented in the polyclinic electronic medical records (EMR), were the target participants. They were screened by the physicians and EMR to identify those who experienced an acute gout exacerbation within the preceding 3 months. They fulfilled diagnostic criteria for gout based on the ACR‐EULAR 2015 criteria^32^ before they were invited by the principal investigator (MM) to participate in the data collection. They were able to speak and read English, as the interviews and discussions were conducted in this common language in Singapore. Patients with any known disability or impairment, which rendered them incapable of providing informed, consent were excluded.

#### 2.5.2 Inclusion and exclusion criteria for PCPs

PCPs were physicians currently providing primary care services at the polyclinics. Those who self‐reported to have ever treated patients with gout were invited.

### 2.6 Purposive sampling

Patients were purposively sampled across diverse age groups and ethnicities to gather a wide range of perspectives. This will optimize the future utility of the PDA by a spectrum of patients in the local community.

PCPs from various seniority and qualification levels were invited. This was a deliberate effort to capture their views based on their years of practice and personal experiences in managing a wide variety of patients with gout.

### 2.7 Topic guide

A topic guide was used through the IDIs and FGDs (Appendix S1, S2). It included questions on patients' and PCPs' perspectives towards the content, format and usability of the PDA. It also covered broader aims of the study to explore the participants' perceptions about gout, its treatment options, and opinions on SDM, which will be covered in subsequent papers.

### 2.8 Questionnaire

The standardized questionnaire for patients recorded their basic demographics and clinical data. Another questionnaire recorded the physicians' basic demographics, seniority at place of practice, length of practice in primary care and qualifications.

### 2.9 Recruitment

The health‐care workers, primarily physicians, nurses, and pharmacists at the study site, were briefed to identify and invite potential patients during clinical consultations. Potential patients were given copies of the prototype PDA and participant information sheet (PIS). Verbal consent was taken to allow patients to be contacted. The screening of potential patients was performed by MM. Sampled patients were contacted, and interviews or discussions arranged. Patients had at least 1 week to read through the PIS and prototype PDA. First three participants underwent IDIs to allow the Principal investigator to develop a coding framework, review and enhance the lineup of questions in the topic guide and to add sub‐questions to trigger interactions in the subsequent FGDs. The FGDs were intended for group discussion and interaction for collective views on the content, design and layout of the PDA. Data collection continued till data saturation was reached, and no new themes emerged from the IDIs and FGDs.

PCPs from the study site were invited during staff meetings to participate. PCPs from other public and private primary care settings were invited purposefully. PCPs had 1 week to read through the PIS and prototype PDA. Data collection continued till data saturation was reached.

### 2.10 Interviews/Discussions

On the day of interview or discussion, participants were again briefed on the study procedure and clarified their queries before endorsing the written informed consent. Demographics were captured in the questionnaire. Patients were separated from PCPs. The IDIs and FGDs were conducted in a quiet room of the polyclinic, which lasted 45‐60 minutes. MM conducted all the IDIs and FGDs, with assistance from other co‐investigators to take field notes. The participants were anonymized using code names. The interviews and discussions were audio‐recorded, transcribed, audited for accuracy (by MM and AL) and rectified.

### 2.11 Data analysis

The investigators (MM and AL) first read and reread the data for familiarization. They then coded the IDIs independently via inductive approach, labelling units of data, by phrases or lines, with codes. After completion of the open coding of the first three IDIs, consensus on the coding framework was reached between the two investigators. This framework was then applied to the subsequent six FGDs and codes expanded or modified after each deliberation. The data were coded using the NVivo‐12 software. The data collection was terminated when ideas were deemed by MM to be saturated after repeated reviews and iterations of codes.

The coded data were inductively grouped into emergent themes that were further categorized under the headings of content, design and perceived utility of the PDA prototype. Quotes were selected to illustrate the themes in reporting the results. The investigators (MM and AL) were aware of their personal biases when analysing the content and engaged in continuous iterations. MM, who was the main facilitator for all the IDIs and FGDs, is a trained family medicine physician, who worked at the study site. AL, who helped MM with data analysis, was a medical student attached to the study site. MM and AL held a constructivist research paradigm.

The consent forms, recordings, transcripts, questionnaires, coding, coding framework and field notes were maintained in secure archives to establish a clear audit trail.

## 3 RESULTS

### 3.1 Characteristics of participants

The participation rate was 30% amongst all invited patients and 73% amongst invited physicians. A total of 26 participants were recruited and participated. They included 15 patients with gout over two IDIs and three FGDs, and 11 PCPs over one IDI and three FGDs. Patients were mainly men, Chinese and were treated with Allopurinol (Table 1). We did not find any differences in opinion based on age or ethnicity. Table 2 shows the characteristics of the PCPs, including those with basic and postgraduate qualifications in Family Medicine, and the level of seniority at the workplace. We did not find any differences in opinion based on qualification or level of seniority.

**TABLE 1:** Patient demographics

	N (% of participants)
Age range	23‐69
Sex	
Male	14 (93%)
Female	1 (7%)
Ethnicity	
Chinese	11 (73%)
Malay	3 (20%)
Filipino	1 (7%)
Duration of gout (y)	0.5‐30
On Allopurinol	12 (80%)

**TABLE 2:** Physician demographics

	N (% of participants)
Age range	30‐44
Sex	
Male	6 (55%)
Female	5 (45%)
Ethnicity	
Chinese	8 (73%)
Indian	3 (27%)
Qualifications	
MBBS	3 (27%)
MD	1 (9%)
GDFM	4 (36%)
MMed	3 (27%)
Number of years as a doctor	5‐20
Seniority at place of practice	
Consultant	1 (9%)
Family Physician	5 (45%)
Resident Physician	4 (36%)
Medical Officer	1 (9%)

Abbreviations: GDFM, Graduate Diploma in Family Medicine; MBBS, Bachelor of Medicine and Bachelor of Surgery; MD, Doctor of Medicine; MMed, Master of Medicine in Family Medicine.

The content, design and perceived barriers/utility of the PDA were categories inducted from the emergent themes (see Table 3 for final coding tree). These aspects of the PDA are facilitating the ‘decision support’ focused on the ‘decisional needs’ of patients as per ODSF.

**TABLE 3:** Final coding tree

Content Understanding the information Treatment options available Language used in PDA Adequacy of information Doctors' want for more information Patients' satisfaction with information Statistical Presentation (Numbers) Doctors' perception of numbers Patients' perception of numbers Preference for particular treatment options (Doctors) Preference for Allopurinol Unfamiliarity with Febuxostat and Probenecid Design Layout Lack of illustrations Systematic but cramped Format: Print versus Digital Digital PDA benefits Serving needs of users Perceived barriers/utility Challenges with SDM Time constraint Suggestions for challenges Preconsultation use of PDA SDM over few consultations

### 3.2 Content

#### 3.2.1 Understanding the information

Both patients and PCPs understood the PDA, especially with regard to the treatment options. Patients reverted that the one‐page summary of treatment options raised their awareness of the available therapeutics.

I think it is good that it shows all the different treatment options. It is in one page and at one glance, they will know what they are getting in terms of each treatment option. I think for patient's choice, it is good. 23‐year‐old Chinese male patient

I like the table behind, the comparison between all the different treatments. I think it is a nice summary, will be good to use this to go through with my patients. 35‐year‐old Chinese male Family Physician

Both patients and PCPs accepted the level of English used in the PDA. However, some felt that older patients could face difficulty understanding its content.

I think a handout like this would be useful for the younger age group. No disrespect to the older ones, it might more difficult for them to understand when they read this. 27‐year‐old Malay male patient

I think the English is simple enough to understand. There are not too many steps, and it is not too overwhelming. 30‐year‐old Chinese female Family Physician

Given the multiethnic population of Singapore and the older age group of patients being served in the polyclinic, both patients and PCPs felt that the PDA should be available in multiple languages.

I think it should be in other languages. In my clinic setting, I have a lot of patients who can only read Chinese, Malay or Tamil, and not English. Having (it) in other languages may help. 35‐year‐old Chinese male Family Physician

#### 3.2.2 Adequacy of information

Patients reported adequacy in the amount of information in the PDA.

Yes, one page is not too long. The long one people would not read. 67‐year‐old Chinese male patient

This perspective contrasted with the PCPs' suggestion of including more information in the PDA. PCPs felt that it should include more details on the pathophysiology of gout, target uric acid levels, complications of gout and differentials for gout.

I realized this PDA did not include serum uric acid target levels. Is there any particular reason? Because usually we mention it in the consult. 35‐year‐old Chinese male Resident Physician

Where are the complications of gout? What are the complications of gout? 32‐year‐old Indian male Family Physician

**FIGURE 3:**
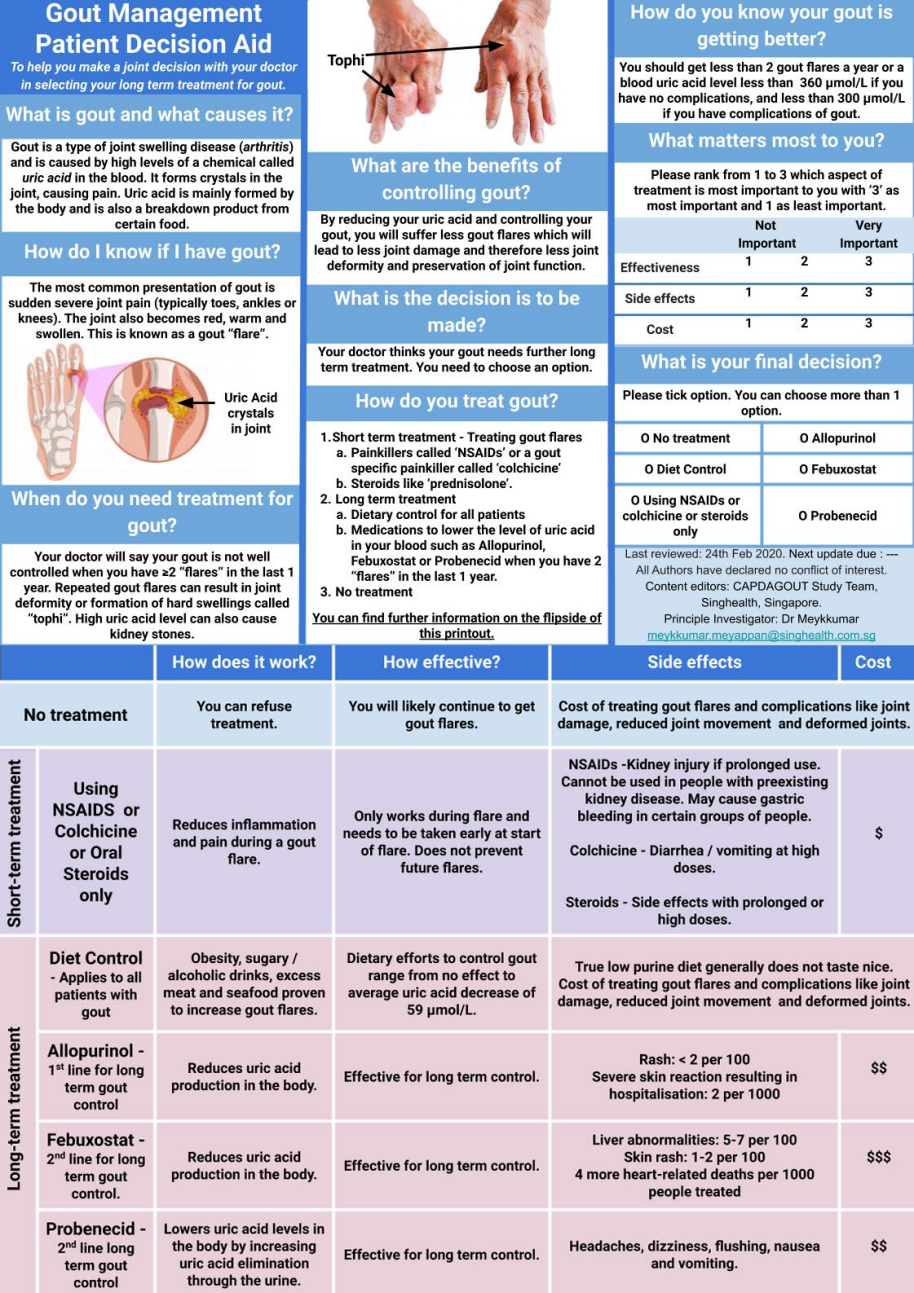
Front page and back page of edited DA

The content of the PDA was expanded to include information on uric acid target levels and pictures of tophi as requested by participants (Figure 3).

#### 3.2.3 Statistical presentation (numbers)

PCPs and patients differed in their perspectives on the use of numbers to present the probability of treatment adverse events. PCPs felt that the numbers might scare patients or that patients were indifferent to statistics. On the contrary, patients felt numbers conveyed a more accurate picture. They were more relieved when they saw the low rates of side‐effects and generally expressed acceptance of the numbers.

I would actually like more statistics to know how effective this drug is exactly. 23‐year‐old Chinese male patient

I think different patients interpret numbers differently. So it depends on the patient, if they want to know the exact risk I will quote it to them or otherwise I will just use the word “rare”. 35‐year‐old Chinese Male Family Physician

I'm happy when I see the numbers because it gives us a value of calculated risk. 39‐year‐old Filipino Male Patient

Do you think if we give numbers, it will make a difference? MM (Interviewer)

Not really. 32‐year‐old Indian Male Family Physician

The statistics on the medication side‐effects were retained in the revised PDA (Figure 3). The effectiveness of the drugs was described in prose rather than numbers for ease of explanation.

#### 3.2.4 Preference for particular treatment options

Few PCPs did not want the inclusion of alternative long‐term urate‐lowering medications to Allopurinol. They felt that Febuxostat and Probenecid should be represented as 2nd line agents in the PDA.

Allopurinol is still the first line, and I would not switch to a second line drug just because the patient asked for it. 44‐year‐old Chinese Male Consultant

We have never started on Febuxostat or Probenecid, and they are not very common medicines to begin with. 30‐year‐old Female Chinese Medical Officer

The study team decided to keep the options of Febuxostat and Probenecid in the revised PDA, (Figure 3) but labelled them as 2nd line options as per current guidelines.

### 3.3 Design

#### 3.3.1 Layout

Both PCPs and patients commented that the PDA layout was neat, systematic, but cramped.

It has a very neat layout. I like it because it is systematic, but I have to say that at one glance it does certainly look busy. 44‐year‐old Chinese Male Consultant

Both PCPs and patients found the PDA too ‘wordy’ and preferred more pictures for illustrations instead.

I don't think I will be reading it because it is a bit too wordy. 23‐year‐old Chinese Male Patient

Looks a little bit wordy, so some patients may not really read it in details and just skim over it. 35‐year‐old Chinese Male Family Physician

Patients perceived that having ‘scary’ pictures of gouty tophi would help other users understand the perils of uncontrolled gout.

Honestly when I was younger I took it very lightly but I think they should be shown more deformities to frighten them a bit. 66‐year‐old Malay male patient

Illustrations were added to the revised PDA (Figure 3) to assist in showing the pathophysiology of gout and the possible severity of uncontrolled gout.

#### 3.3.2 Format: print vs digital

Both PCPs and patients agreed that PDA in print would serve its function but suggested better portability with digital PDA. One PCP suggested having a QR code to access the digitalized content. Some felt that either format would cater to different patient profiles. What mattered to them was whether the PDA served the needs of the users.

I think what's more important is what suits the patient. If the patient is one who is very internet savvy, whips out his phone and finds anything on Mr Google, then websites would be more suited for such a patient. If the patient is a little old fashioned, likes hard copy materials to flip through and read at his own leisure, then a hard copy would be more suitable. 44‐year‐old Chinese male consultant

### 3.4 Perceived barriers/utility

Both PCPs and patients recognized the time constraint present during consultations to use the PDA for shared decision making.

In all fairness, unless you come to a private doctor, the polyclinic only has a few minutes with you. There are so many people and the queues are so long. I think honestly the doctor should have more time with us but they cannot take too long if not the waiting time will be longer. 67‐year‐old Chinese male patient

Patients opined that the PDA could be used as a tool to understand the gout‐related information before the consultation. They could then ask the physicians more useful questions during the short consultation period.

If you just talk, only 5% will go in. This kind of information is good especially if you have hard copies, it is good for reference. 58‐year‐old Chinese male patient

I really admire this PDA because there is a lot of info that the patient can gather from the PDA which the physician cannot give within the short consultation time. There is a lot of information. It is very, very beneficial to the patients. 34‐year‐old Indian male Resident Physician

I would definitely read this before the consult so that I can go in there and ask all the questions I want to ask. It would be a lot quicker. 23‐year‐old Chinese male patient

PCPs would consider using the PDA over a few consultations.

With every chronic disease, it is not done in one sitting. It has to be broken up into small bits every time the patient comes back. 31‐year‐old Chinese Female Resident Physician

One PCP commented that the PDA could serve as a medicolegal document to record the discussion with the patients on the options and risks.

I think this will be good to have and because it's part of the medical legal process. It you did go through this and by going through this they should understand the risks involved in certain treatment and if a complication arises in patient and patient makes complaints, at least It's forgiven that you did go through all the complications with the patient. 35‐year‐old Chinese male Family Physician

PCPs and patients were ready to try out the PDA.

Yes I would give it a try and I would see how things go. Whether it is helping the patient to understand things better. I'll definitely give it a try. 36‐year‐old Indian Male Family Physician

## 4 DISCUSSION

Patients and PCPs differed on their opinions of the amount of information to be included, and the use of numbers in the PDA, but they concurred on the understandability of the PDA, its lack of illustrations, systematic but cramped display, time constraint and suggestions to overcome the barriers. Both concordant and contrasting views of the participants were considered in developing the 2nd version of the PDA (Figure 3). This study thus shows that both patients and physicians can be involved in the prototype development of a PDA.

The volume of information to be included in the PDA was a frequent source of contention in the study. PCPs wanted more information to be included while patients were satisfied with the volume of information. This difference might have its roots in the inherent information asymmetry present in the physician‐patient relationship.^33^ Studies have shown differences in the informational needs of physicians and patients.^34^, ^35^, ^36^, ^37^ Physicians usually have broader and in‐depth knowledge of the disease and treatment options compared to a patient, such that they might subconsciously overload patient with too much information. In contrast, patients only need information relevant to their decision making. The study team, therefore, recognized the importance of training the PCPs in SDM so that aside from providing concise information, they should strive to elicit the values and preferences of the patient. Pertinent information such as uric acid target levels was included in the PDA (Figure 3).

Physicians and patients are known to differ in their opinions on the use of statistics in PDAs.^38^, ^39^ Inclusion of quantitative information in a PDA has been shown to improve patients' accuracy of risk comprehension and lead to better‐informed decision‐ making.^24^ Despite this, physicians perceive that numbers were not relevant to patients.^24^ This might be rooted in their training based on the paternalistic model of care. Physicians could have underestimated the ability of patients to handle and interpret numbers and risks. In addition, most of the patients in this study were well educated. This again highlighted the importance of training physicians in SDM. Hence in the revision of the PDA, the statistics on the medication side‐effects were retained in the treatment matrix. Prose was used to describe the effectiveness of the drugs. Numbers in the PDA would be complemented with visual aids in a future digital version of the PDA. Visual aids have been shown to aid the understanding of numerical data,^40^ which concurrently assuage physicians' concerns about patient's numerical literacy.

One salient finding was the PCPs' preference for displaying Allopurinol as the only urate‐lowering medical therapy option in the PDA. They were not in favour of including Febuxostat and Probenecid as alternative urate‐lowering medications. Unfamiliarity with the use of the drugs, lack of access to Febuxostat in primary care, desire to follow specific guidelines advocating the use of Allopurinol as the first‐line therapy and the physicians' perceived higher efficacy of Allopurinol over the other two agents were commonly cited reasons. This finding again highlights the conspicuous lack of SDM and PDAs in current clinical practice. SDM focuses on the patient's autonomy in decision making,^41^ which is often overlooked by their attending physicians. Guidelines from the local Agency for Care Effectiveness (ACE),^42^ the European League Against Rheumatism (EULAR)^43^ and those from the American College of Rheumatology (ACR)^44^ differ in their recommendations of first‐line medications for urate‐lowering therapy, which could have resulted in variable prescribing preference of the physicians. What is important is to induct the physicians to the concept of providing treatment options to patients in SDM, and bringing their attention to the merits, risks and availability of each option. The patient is the recipient of the treatment, not the physician. It is their right to know the alternative therapeutic options.

The perception of the increased time required to go through a PDA is contrary to results from studies,^24^, ^45^, ^46^, ^47^ which found no or minimal increase in the consultation time with SDM. This remains to be tested in Singapore, where mean consultation time ranged from 10 minutes for polyclinics to 15.8 minutes for private general practitioners.^48^ Suggestions were offered by PCPs and patients to overcome this barrier. Patients can read the PDA before or after the consultation and should not be rushed to make a decision within stipulated consultation time. The process of SDM can be spread over multiple consultations. Nurses, pharmacists and dieticians can be trained in SDM to support the PCPs. This multidisciplinary approach in SDM is likely to be scaled up, given increasing complexity of managing patients with multiple co‐morbidities.

Cultural optimization, in the development of this PDA, underpins the strength of the study. Cultural adaptation has been used to develop PDAs to fit local cultural contexts.^49^, ^50^, ^51^ However, cultural optimization brings this process one step further by facilitating the development of a PDA de novo. This study has demonstrated that physicians and patients can work together to refine a PDA, which is specially designed for use by local patients and health‐care professionals.

To enhance the research rigour, physicians and patients of different profiles are purposively invited as participants as a deliberate attempt to garner perspectives from a spectrum of end‐users of the PDA. This would help reach out to more patients and physicians to use the PDA when it is implemented in routine clinical services. During the FGD, we had kept the number of participants to five to six, so that the trained facilitator could moderate the discussion to ensure adequate time for each participant to speak up. The facilitator referred to the length of speech time from the audio‐recordings to assess the contribution from all the participants. We analysed the qualitative data from both the physicians and the patients concurrently, using reflexivity throughout the study, to ensure that balanced views are presented from both stakeholders.

Limitations exist in this study. Singapore has a dual primary health‐care system. Patients and physicians were recruited from a public primary care institution in Singapore. Patients and physicians from private clinics might differ in their opinions and utility for the PDA. Nonetheless, the PDA will be available for the latter to adapt it to their setting.

Patient participants were majority males in this study, compatible with the demographic profile of local patients with gout. Patients should ideally have been included in the steering committee to conceptualize the first draft of the PDA. With the formation of patient advocacy and support group for research, their involvement will be stepped up in future PDA development.

Member checking would be ideal to ensure appropriate interpretation of the qualitative data. However, this procedure was not carried out as it was not specified in the approval protocol that participants would be contacted again. English was used in the IDI and FGD. While the language medium does not reach out to the non‐English speaking participants in Singapore, the plan was for the translation of the English edition to other local languages once the feasibility of the DA is proven in the next phase of the study.

Non‐physician health‐care providers were not involved as physician‐participants. Only a small number of nurses and pharmacists currently hold prescription rights in our setting. While PCPs were purposively recruited as participants in this early phase of DA development, views from the other health‐care providers will be gathered in the subsequent implementation phase.

In conclusion, both contrasting and convergent views of patients and physicians helped facilitate the development of a novel gout PDA culturally optimized to Asians in a developed community. The PDA will be introduced in clinical practice to evaluate its effectiveness in enhancing SDM and patient's adherence to urate‐lowering therapy. Physician deficits in understanding the principles behind SDM and PDAs will need to be addressed in the next phase of field‐testing this PDA.

## CONFLICT OF INTEREST

The author reports no conflicts of interest in this work.

## AUTHORS' CONTRIBUTIONS

Principle Investigator: Dr Meykkumar Meyappan: Conception of study, applying for grant and IRB approval, setting up team, conducting interviews, transcribing, coding, analysis, writing manuscript, submission to journal. Co‐investigators: Medical Student: Loh Wei Siong Aaron: Note taking during interviews, transcribing, coding. Nurse: Tan Li Yen. Pharmacist: Tan Sheng Feng Ian. Dietician: Ho Pey Ying. Multidisciplinary team for conception of 1st PDA, assistance during interviews. Rheumatologist: Dr Poh Yih Jia: Input for prototype PDA. Clinical Assoc Prof Ngiap Chuan: Conception of study, training of team on qualitative methods, guidance on project, vetting grant and manuscript.

## ETHICAL APPROVAL

The study was approved by the SingHealth Centralised Institutional Review Board (CIRB reference 2019/2105).

## INFORMATIVE

In this paper, we show how patient and primary care physician input enabled the co‐creation of a culturally optimized novel patient decision aid (PDA) for gout treatment. This study is significant as we are not aware of any gout treatment PDA available globally. Such a PDA can potentially overcome the barriers to better management of gout.

## Supporting information

Appendix S1

Appendix S2

## Data Availability

Data available on request due to privacy/ethical restrictions.
