# Supplementary material for: Development of a novel gout treatment patient decision aid by patient and physician: A qualitative research study
Source: Health Expect. 2021 Jan 12;24(2):431–43. doi: 10.1111/hex.13184 (PMC8077153; doi:10.1111/hex.13184)
Supplement: Supplementary file 1 — Appendix S1 [file HEX-24-431-s002.pdf]

TOPIC GUIDE

CAP-DA-GOUT STUDY

Aim of this Interview / focus group discussion -

Among primary care physicians (PCP)s who treat patients with gout, we aim to understand their gout treatment plan and goal; their views on the involvement of the patient in decision making in selecting their treatment options and the use of a patient decision aid (PDA) prototype to facilitate shared decision making to manage gout.

The questions to be raised during the interviews:

1. In primary care physicians who see patients with gout, what is their understanding of gout's treatment plan? What do they aim to achieve in such treatment? What is their view of long term treatment to control gout? How much do they know about the side effects, benefits and costs of the various treatment options?

2. What are their experiences of a typical consultation for a patient with acute gout exacerbation? What do they do when they see patients with known gout but without any acute exacerbations? What is their perception of patient involvement in decision making in selecting their treatment options? How much do they know about shared decision making and the use of patient decision aid (PDA)?

3. What are their priorities when discussing treatment options with the patient?

4. What are their views on the prototype PDA developed for gout treatment? What are the additional information that they need, if any, in discussing gout treatment? Will they use such a PDA for gout and why?
