# Supplementary material for: Development of a novel gout treatment patient decision aid by patient and physician: A qualitative research study
Source: Health Expect. 2021 Jan 12;24(2):431–43. doi: 10.1111/hex.13184 (PMC8077153; doi:10.1111/hex.13184)
Supplement: Supplementary file 2 — Appendix S2 [file HEX-24-431-s001.pdf]

TOPIC GUIDE

CAP-DA-GOUT STUDY

<u>Aim of this Interview / group discussion -</u>

Among patients with known gout, as reported by their doctor, we would like to understand their views of gout, treatment, perceived gout control, experience of the patient-doctor consult, decision making in selecting treatment options, and views for a prototype patient decision aid (PDA) for gout treatment.

<u>The questions include:</u>

1. In Asian patients with recent gout attacks, what is their understanding of gout and its treatment? What do they understand about gout control? What is their view of long term treatment to control gout?

2. What are their experiences of their doctor consultation during their recent gout attack? What do they think about making decisions together with their doctor in selecting their treatment options?

3. What will enable the patients to make informed decisions on their treatment? What are their priorities and concerns?

4. What is their opinion on the prototype PDA (will be shown to patients) developed for gout treatment? Is there any additional information that they wish to find out? Is it easily understood? Does it meet their needs in selecting their long term treatment?
